# Roles of Probiotics in Reduction of Neonatal Jaundice in Term Newborns

**DOI:** 10.14789/jmj.JMJ21-0044-OA

**Published:** 2022-02-16

**Authors:** IRENA SANTOSA, HIROMICHI SHOJI, SHIGERU ITOH, TOSHIAKI SHIMIZU

**Affiliations:** 1 Department of Pediatrics and Adolescent Medicine, Juntendo University Graduate School of Medicine, Tokyo, Japan; 1 Department of Pediatrics and Adolescent Medicine, Juntendo University Graduate School of Medicine, Tokyo, Japan; 2 Sakuradai Maternity Clinic, Tokyo, Japan; 2 Sakuradai Maternity Clinic, Tokyo, Japan

**Keywords:** caesarean section, hyperbilirubinemia, jaundice, probiotics, transcutaneous bilirubinometer

## Abstract

**Objective:**

the primary objective was to examine the effect of Bifidobacterium on decreasing the bilirubin level in term neonates delivered by Caesarean Section (CS).

**Materials and Methods:**

A total of 153 healthy term neonates delivered by CS were included in this study and were divided into the non-probiotic group (n=99) and probiotic group (n=54) based on the history of probiotics administration. There were no infants who underwent phototherapy. A total of 20 doses of probiotics were given orally from the first day of life. The transcutaneous bilirubin (TcB) levels were measured every day for the first 5 days of life. Data of each infant and mother were gathered from medical records.

**Results:**

The bilirubin level per day (day-1 to day-5) in the non-probiotic group was no different from the probiotic group. Differences in bilirubin level between day-5 and day-1, and also between day-5 and day-2 were not different between the two groups. There was a significant (p = 0.03) body weight gain in the probiotic groups with a mean of 36.09 ± 8.23 gram/day. No obvious adverse reactions were seen in both the non-probiotic group and probiotic group.

**Conclusions:**

Our findings suggest no significant effects of probiotics on lowering bilirubin levels in the first five days of life. Also, probiotics have a positive effect on body weight gain in healthy term infants, and it is safe to be given to newborns.

## Introduction

Approximately 60% of term and 80% of premature infants have elevated total serum/plasma bilirubin (TSB) levels, which results in neonatal jaundice in the first week of life^1)^. Jaundice is one of the most commonly encountered symptoms during the neonatal period and one of the leading causes of admissions in newborn nurseries throughout the world^2)^. Jaundice in newborns occurs due to high levels of unconjugated bilirubin in the blood that causes by ABO/Rhesus incompatibility, G6PD deficiency, infections, prematurity, and metabolic disorders^3)^. The etiology of neonatal hyperbilirubinemia is multifactorial, such as physiological factors, isoimmunization, and environmental factors. One of the environmental factors is the dysbiosis or lack of the microbiota in the guts is considered to be one of the pathogenic factors for neonatal jaundice^4, 5)^.

At birth, infant’s gut is considered sterile and the early development of intestinal microbiota in neonates born vaginally starts at birth due to the acquisition of organisms from the vaginal microbiota, other maternal sources, and environmental sources. Therefore, newborns delivered by Caesarean Section (CS) have a bigger risk of dysbiosis and the primary gut flora may be disturbed for up to six months. Also, the intestinal microbial diversity is lower in the first two years of life in infants delivered by CS^6)^.

Physiological jaundice is self-limiting and does not need treatment. However, in some cases, bilirubin levels can reach more than 20 mg/dL, which requires treatments and may result in complications such as kernicterus and damage in the neurological system. Phototherapy is still the main therapy for significant jaundice, but this therapy is also associated with complications such as erythematous rashes, diarrhea, temperature instability, and dehydration. Also, the newborns who underwent phototherapy were separated from the mother and may interfere with the process of lactation. Therefore, prevention and other treatment of neonatal hyperbilirubinemia are very important^7)^.

Recently, some evidence suggests that gut microbiota plays a critical role in maintaining metabolic and immune health, synthesis of vitamins, renewal of epithelial cells, fat storage, and maintaining intestinal barrier integrity. Probiotic supplementation can reduce bilirubin levels by several mechanisms such as reducing the enterohepatic circulation by the regulation of intestinal flora and also by increasing meconium evacuation^8)^.

Probiotics are being studied for their potential benefit in being a treatment of indirect hyperbilirubinemia, but the potential of probiotics as prevention has not yet been clarified. The early gut microbiota is often dominated by Escherichia. Clostridium, Bacteroides, and Bifidobacterium which is low in infants delivered by CS. Therefore, in this study, the primary objective was to examine the effect of Bifidobacterium on decreasing the bilirubin level in term neonates delivered by CS.

## Methods

### Subjects

A total of 157 healthy term neonates delivered by CS in Sakuradai Maternity Clinic (January 2017 - December 2019), who was born at 36-40 weeks gestational age were included in this study. A total of 4 infants underwent phototherapy in both probiotic group (n=1) and non-probiotic group (n=3), and these 4 infants were excluded from the study. Parents received information regarding probiotics and their benefits. Neonates whose parents agreed to the administration of the probiotic (n=54) were included in the probiotic group (n=99) and those whose parents refused will be included in the non-probiotic group (n=153). There were no infants who underwent phototherapy. This study was carried out by the opt-out method of the clinic website, and it was approved by the Institutional Review Board of Juntendo University Faculty of Medicine on 21 January 2019 (Registration Number: 18-159; Approval Number: 2018162).

### Methodology

The Probiotic group was treated with a total of 20 doses (daily doses: 6 drops containing 10 x 10^8^ CFU) of commercial probiotic products (*Bifidobacterium animalis subsp*. *lactis* BB-12, Bean Stalk Snow Co.,Ltd, Tokyo, Japan), which were given orally from the first day of life until day 20. The TcB levels were measured by using the transcutaneous bilirubinometer (JM-103, Konica Minolta, Osaka). TcB levels were measured every day for the first 5 days of life. Data of each infant’s bilirubin levels, anthropometric measures, type of feeding, and mother’s anthropometric measures were gathered from medical records.

### Statistical analyses

The normally distributed variables (checked by a 1-sample Kolmogorov-Smirnov test) were compared using an independent sample t-test between the groups: Bilirubin levels on day 1 until day 5; Birth Weight; Body Weight gain; and one-month-old Body Weight. A Mann-Whitney U test was used to compare variables between the groups for variables that were not of a normal distribution. The chi-square test was used to compare between categorical variables in the two groups (gender, parity, and type of feeding). A p-value of <0.05 was considered statistically significant. All statistical analyses were conducted using GraphPad Prism version 9.0.0 (GraphPad Software, La Jolla, California, USA).

## Results

The total number of newborns included in this study was 153, with 54 newborns received probiotics and 99 were taken as controls. Table 1 summarizes the demographic of infants and type of feeding (first five days of life). Comparison between the groups showed that there were no significant differences with respect to gender, parity, and type of feeding (breast milk, mix of breast milk and formula milk, and formula milk only).

**Table 1:** Demographic and Characteristics of Infants and Mother^a)^

		Control(n=99)	Probiotic(n=54)	Significance^b)^
Gender				
	Male	56 (57%)	32 (59%)	P > 0.05
	Female	43 (43%)	22 (41%)	
Parity				
	First Child	33 (33%)	14 (26%)	P > 0.05
	Not	66 (67%)	40 (74%)	
Type of Feeding				
	Breast Milk	29 (29%)	18 (33%)	P > 0.05
	Mix	70 (71%)	35 (65%)	
	Formula Milk	0	1 (2%)	

^a)^ Data are shown as the number (percentage) of subjects. ^b)^ A p-value of <0.05 was considered statistically significant.

**Figure 1:**
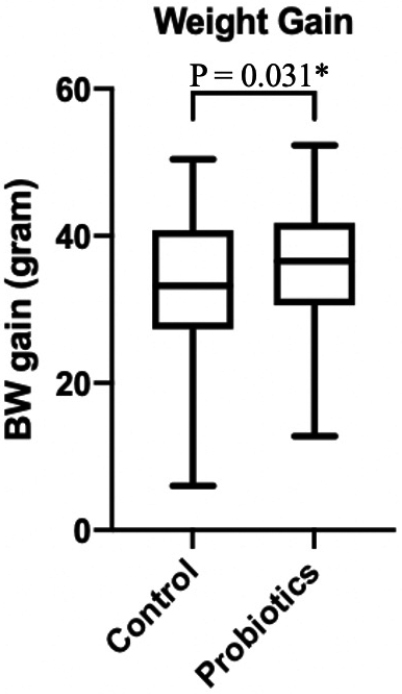
Comparison of weight gain (gram/day) *BW gain: Body weight gain **Compared using student t-test and p-value of <0.05 was considered statistically significant.

Probiotic supplementation caused a significant difference (p = 0.031) in the weight gain of probiotic groups (Figure 1). The mean weight gain in probiotic groups are 36.09 ± 8.23 gram/day compared to newborns in control groups is 32.86 ± 9.05 gram/day. Other anthropometric data of the newborns in the two groups were not different significantly. Anthropometric data such as body weight, body length, and head circumferences are summarized in Table 2. Also, a comparison of maternal BMI and gestational age were shown in Table 2.

**Table 2:** Anthropometric Data of Infants and Mother^a)^

		Control (n=99)	Probiotic (n=54)	Significance^b)^
Mother BMI^c)^		21.18 ± 2.54	21.07 ± 2.48	P > 0.05
Gestational Age		38.56 ± 1.12	38.57 ± 1.18	P > 0.05
*At Birth*				
	Body Weight (gram)	2991 ± 324.2	3001 ± 352.9	P > 0.05
	Body Length (cm)	48.93 ± 1.48	48.97 ± 1.93	P > 0.05
	Head Circumference (cm)	34.02 ± 1.10	34.25 ± 1.21	P > 0.05
*At One Month*				
	Body Weight (gram)	4058 ± 419.6	4198 ± 436.8	P > 0.05
	Body Length (cm)	54.16 ± 1.82	54.12 ± 1.82	P > 0.05
	Head Circumference (cm)	36.39 ± 1.16	36.55 ± 1.13	P > 0.05
Weight gain(gram/day)		32.86 ± 9.05	36.09 ± 8.23	P = 0.03

^a)^ Data are shown as mean ± standard deviation. ^b)^ A p-value of <0.05 was considered statistically significant. ^c)^ BMI: Body Mass Index

**Figure 2:**
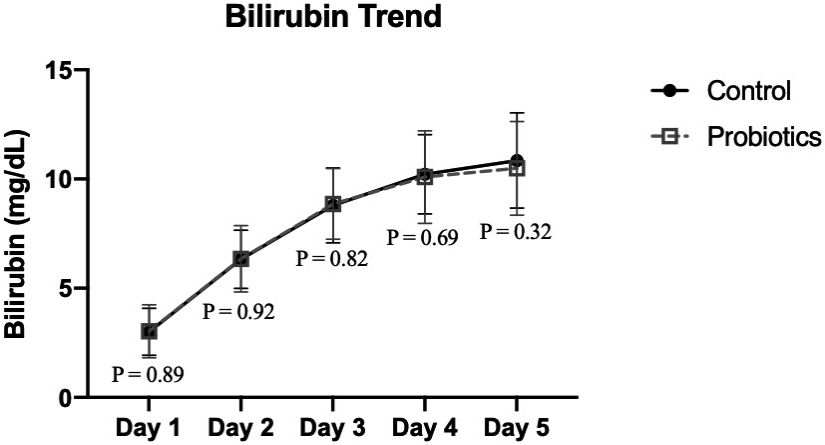
Bilirubin trend from Day-1 to Day-5 *Compared using student t-test and p-value of <0.05 was considered statistically significant.

Figure 2 shows the mean bilirubin level trend from day 1 to day 5. The mean bilirubin level on day 1 is 3.00 ± 1.08 mg/dL in the control groups and 3.03 ± 1.21 mg/dL in probiotic groups, which showed no differences between both groups. On day 2, the mean bilirubin level is increasing almost 2-fold from day-1 (6.33 ± 1.34 mg/dL in control groups and 6.35 ± 1.52 mg/dL in probiotic groups). On day 5, the bilirubin levels in both groups still increasing with a mean of 10.85 ± 2.18 mg/dL in the control groups and 10.49 ± 2.14 mg/dL in probiotic groups.

The increase of bilirubin level from day 1 to day 5 was found in both groups. On day 5, after the last dose of probiotics was given, the bilirubin level between probiotic and control groups showed no significant differences. Also, there are no significant differences in the increased rate of bilirubin level on day 5-day 1 and day 5-day 2 between the two groups (Table 3).

**Table 3:** Comparison of Bilirubin Level^a)^

Bilirubin Level (mg/dL)	Control (n=99)	Probiotic (n=54)	Significance^b)^
Day 5 - Day 1	7.85 ± 1.93	7.45 ± 2.19	P > 0.05
Day 5 - Day 2	4.53 ± 1.69	4.14 ± 1.93	P > 0.05

^a)^ Data are presented as mean ± standard deviation. ^b)^ A p-value of <0.05 was considered statistically significant.

All of the infants enrolled in this study didn't have any Adverse Events (AEs), which is defined as illnesses or sign and/or symptoms that occurred or worsened during the course of the study. These were assessed based on infant's medical record.

## Discussion

Neonatal hyperbilirubinemia is a common problem in newborns, can be classified into physiological and pathological jaundice. Jaundice is usually a transient condition, but in pathological jaundice, the increase of bilirubin level is persistent and may reach dangerous toxic levels leading to kernicterus. Therefore, it should be treated timely. Neonatal jaundice is caused by an undeveloped intestinal microbiome and increased enterohepatic circulation contributes to an increase of plasma bilirubin level in the first days of life^9)^.

The increase of enterohepatic circulation is due to the high content and activity of *β*-glucuronidase (*β*-GD). At the brush edge of the intestinal tracts, *β*-GD deconjugates the bound bilirubin into unbound one and glucuronide. The unbound bilirubin is reduced by intestinal microbiota to a series of urobilinogen and reabsorbed by intestinal cells to the liver via the portal vein and this causes a boost in the enterohepatic cycle. Also, newborns lack the intestinal microbiota which helps to reduce the unconjugated bilirubin^4, 10)^.

Probiotics are a living micro-organism which potential benefits have been increasing since the last two decades^8)^. A study showed that probiotic supplementation can affect the neonatal hyperbilirubinemia by various potential mechanisms to balance the microbiota dysbiosis and reduce bilirubin level: 1) Promote colonization course; 2) Suppress pathogenic; 3) Stimulate intestinal peristalsis and increase stool frequency which reduce the enterohepatic circulation and inhibit the activity of the enzyme *β*-GD which reducing the degradation of bound bilirubin; 4) Enhance the tight junction; 5) Increase polyamines in the gut to improve the gut maturity^11)^.

Stimulation of the intestinal peristalsis in neonates not only beneficial for lowering the bilirubin level, but gut motility also affects nutrient absorption and one of the major factor impacting the levels of propionic acid. Propionic acid is a short chain fatty acid produced by bacteria in gut, which has been studied as a potential contributing factor to autism. Increased level of propionic acid have been shown to impair brain function in rat models^12)^.

Dysbiosis of the gut microbiome can causes adverse neurological outcomes in later life including cerebral palsy, attention deficit / hyperactivity disorder (ADHD) and reduced in cognitive performance. Also, it was found that children with Autism Spectrum Disorder (ASD) have altered gut microbiota. The gut microbiota may control the central nervous system (CNS) by brain-gut axis^13, 14)^.

The gut-brain axis is a communication system that integrates neural, hormonal, and immunological signaling between the gut and brain^15)^. Although the mechanism how the gut microbiota communicate with the brain is not yet known, but some studies found that infants treated with probiotics have a better neurological outcomes^16-18)^.

One of the popular probiotic strain, Bifidobacterium, can directly metabolize bilirubin which is related to the lower activity of *β*-GD. It is also found that Bifidobacterium can reduce the bilirubin level by increasing stool frequency, decreasing enterohepatic circulation, and lowering intestinal^7, 19)^.

In this study, we observed the effect of a probiotic on lowering bilirubin levels through the first 5 days of life by using the transcutaneous bilirubin meter. The validity of this bilirubin meter has been established by a study in Mongolian neonates as a screening tool for neonatal jaundice in both term and late preterm infants^10, 20)^.

Study by Mutlu et al^21)^. reported that mean total bilirubin levels on day 3, day 5 and day 10 were significantly lower in the probiotic group receiving Lactobacillus Rhamnosus GG. Based on those results, probiotic has effect on lowering bilirubin level and may reduce the risk of hyperbilirubinemia. Also in study by Suganthi et al^2)^., it is found probiotics can lower the serum bilirubin levels significantly in neonates treated with Saccharomyces boulardii. In this study, we recorded the bilirubin level on day 1 until day 5 but there were no significant differences in bilirubin levels at day 1 until day 5 between probiotics and control groups. There were also no significant difference when comparing bilirubin level on day 5 with day 1 or day 5 with day 2.

We preferred to investigate the effect of single strain preparations rather than combined preparations to get the specific results of each strain. The effect of probiotic in this study may be differ from the above studies^2, 9, 21)^, because we used a different strain of probiotics and there were no infants underwent phototherapy in this study. The effectivity of probiotics depends on optimal dose for viability, it means there should be enough or adequate numbers of probiotic strain surviving the barriers such as gastric acid, bile, and competing flora to have beneficial effects^22, 23)^.

In early postnatal period, intestinal flora in newborns are insufficient and infants will start accumulating intestinal microbiota until a stable state is reached. This accumulation process is influenced by environmental factors including nutrition, i.e. breastfeeding versus formula milk^24)^. Nutrition has a major role and alters neonatal intestinal microbiome colonization patterns. Breast milk contains beneficial components, such as lactoferrin, or secretory IgA, which can stimulate the growth of Bifidobacterium species (spp.). Also, human milk contains live bacteria that might contribute to microbiome development in newborns^25)^. Since breast milk contribute in microbiome development, it is assumed that newborns receiving breast milk have lower bilirubin levels. In this study, the differences between the type of feeding is statistically insignificant so this factor won't affecting the result of this study.

Women who were overweight prior to becoming pregnant tended to have a different microbiota than normal weight and obese women^26)^. Since microbiome are transmitted from mother to infants, pre-pregnancy BMI is reported to be associated with microbiota in early postnatal period. However, in this study we found no significant differences in maternal pre-pregnancy BMI of both groups.

In this study, we found that probiotic supplementation caused a significant difference (p = 0.031) in the daily weight gain of infants in probiotic groups. Studies on the use of probiotics (Bifidobacterium and Lactobacillus) suggest that there are association between administration of probiotics with the improved weight gain. This results showed that early modulation of the gut microbiome by giving probiotics supplement is likely to result in increased weight gain^27)^. Not only in healthy term infants, Kitajima et al^28)^. found that very low birthweight (VLBW) infants receiving probiotics also have a better weight gain, as a result of stabilization of the intestinal flora and accelerated feeding schedules.

Studies found that probiotic administration to newborns is safe and have several benefits. Accordingly, administration of probiotics to premature infants also found to be safe and can decrease the risk of death, NEC, and sepsis^28, 29)^. In this study, of 54 newborns received probiotics for the total of 5 days, none of the newborns had any complications related to probiotic administration.

## Limitation

There were only TcB level and no TSB level in this study, because invasive procedure such as blood draw is not permitted on a healthy infants. Also, analysis of intestinal microbiota was not done because there were no stool samples for taken from the subjects in this study.

## Conclusions

There was no significant difference in bilirubin level on day 1, 2, and 5 between probiotic and control groups. Also, we found no significant difference in terms of suppressing the increase of bilirubin level between day 5 – day 1 and day 5 – day 2. *Bifidobacterium animalis subsp. lactis* BB-12 is well tolerated in all infants and there was a significant (p = 0.031) weight gain in the probiotic groups. There were no side effects from the probiotics founded in this study. However, the optimal time to administer probiotics, the strain, dose and duration require more studies.

## Funding

The authors received no financial support for the research.

## Author contributions

The authors confirm contribution to the paper as follows:

Study conception and design: IS, HS; data collection: SI; analysis and interpretation of results: IS, HS; draft manuscript preparation: IS, HS, TS. All authors reviewed the results and approved the final version of the manuscript.

## Conflicts of interest statement

The Authors declare that there are no conflicts of interest.
